# Predicted protein-protein interactions in the moss *Physcomitrella patens*: a new bioinformatic resource

**DOI:** 10.1186/s12859-015-0524-1

**Published:** 2015-03-16

**Authors:** Scott Schuette, Brian Piatkowski, Aaron Corley, Daniel Lang, Matt Geisler

**Affiliations:** Department of Plant Biology, Southern Illinois University, Carbondale, IL USA; University of Freiburg, Plant Biotechnology Schaenzlestr. 1, D-79104 Freiburg, Germany

**Keywords:** *Physcomitrella*, Protein-protein interaction, Interolog, Predicted interactome, Protein network

## Abstract

**Background:**

*Physcomitrella patens,* a haploid dominant plant, is fast becoming a useful molecular genetics and bioinformatics tool due to its key phylogenetic position as a bryophyte in the post-genomic era. Genome sequences from select reference species were compared bioinformatically to *Physcomitrella patens* using reciprocal blasts with the InParanoid software package. A reference protein interaction database assembled using MySQL by compiling BioGrid, BIND, DIP, and Intact databases was queried for moss orthologs existing for both interacting partners. This method has been used to successfully predict interactions for a number of angiosperm plants.

**Results:**

The first predicted protein-protein interactome for a bryophyte based on the interolog method contains 67,740 unique interactions from 5,695 different *Physcomitrella patens* proteins. Most conserved interactions among proteins were those associated with metabolic processes. Over-represented Gene Ontology categories are reported here.

**Conclusion:**

Addition of moss, a plant representative 200 million years diverged from angiosperms to interactomic research greatly expands the possibility of conducting comparative analyses giving tremendous insight into network evolution of land plants. This work helps demonstrate the utility of “guilt-by-association” models for predicting protein interactions, providing provisional roadmaps that can be explored using experimental approaches. Included with this dataset is a method for characterizing subnetworks and investigating specific processes, such as the Calvin-Benson-Bassham cycle.

**Electronic supplementary material:**

The online version of this article (doi:10.1186/s12859-015-0524-1) contains supplementary material, which is available to authorized users.

## Background

A compilation of the physical interactions between the proteins of an organism is known as the protein-protein interactome. It is through these interactions that most biological processes within a living cell are performed [1]. While interactomes can be a composed from many individual published experiments, several high-throughput methods have been developed to rapidly identify interactions between proteins even those with no known function. In doing so, high-throughput methods reveal much of the signalling and communication within the proteomes of yeast, human, fruit fly, and nematode worm [2-5]. With 84% of the proteins interactions experimentally determined, the yeast interactome map is nearly complete [6,7]. However the same is not yet true for the other model organisms, especially plants where high-throughput methods have only recently begun to be employed [8].

To fill in these gaps, predicted interactome maps have been constructed for *Arabidopsis thaliana* (herein referred to as Arabidopsis), rice, coffee, yeast, human, mouse, and fruit fly at the organelle and whole cell levels using computational interolog methodology [6,7,9-13]. The interolog method of predicting protein interactions is based on the hypothesis that conserved orthologous genes encode functionally similar proteins forming similar complexes and signalling pathways [1,14]. This method assumes that functionally similar proteins retain their interacting partners. Using interologs, a predicted protein interactome can be constructed without the extreme expense or time requirement of high-throughput methods, which currently have only been done for selected model organisms. This provisional roadmap of the predicted interactions is by nature of the methodology incomplete, and only contains interactions among proteins that are highly conserved.

The model moss *Physcomitrella patens* is fast becoming a tool for bioinformatics and molecular work due to its key phylogenetic position as sister to the other land plant lineages. *Physcomitrella patens* has a protein-coding genome similar in size to *Arabidopsis thaliana*, but is similar to yeast in efficiency of gene targeting experiments and haploid-dominance of the life cycle, making an interesting and useful molecular genetic tool for plants [15-17]. This moss genome has been sequenced and annotated based on sequence homology to known genes and domains [18,19]. The annotated moss genome is a key tool for research into the evolution of all plant functions. As a bryophyte, this model moss is the placeholder for early land plants on the tree of life, making evolutionary comparisons of biological pathways at the protein interaction level a useful new avenue of investigation.

Presented here is the first predicted PPI for a bryophyte based on the interolog method. Bias and enrichment of gene functions in the predicted interactome were analysed in order to help the user evaluate the utility and interpret the results of this tool. Although derived from many reference organisms, only a few plant and cyanobacterial reference interactions were available for orthology mapping. A confidence value (CV) was assigned to each interaction in order to help determine credibility of predicted interactions. Resolution of the plant specific protein networks, the Calvin-Benson-Bassham (CBB) cycle network, is shown to illustrate the bait and prey methods of capturing functional subnetworks. This tool provides only a conserved eukaryotic skeleton of biological pathways and interactions, but can aid current research and provide the framework for future avenues of interactomic research in plants.

## Methods

### Construction of MySQL database

Creating the merged interaction database was a four-step process beginning with the development of standardized identifiers for each interactor. Secondly, conversions from non-chosen identifiers that exist in four interaction databases (BIND, DIP, BioGrid, and IntAct) to the selected identifiers were found and placed inside SQL databases [20-23].

Third, a Universal Translator program for reference interactomes (see Additional file 1 for source code) was developed to create mappings between the three chosen identifiers, Swiss/Uniprot ID, Entrez Gene ID, and Ensembl Peptide ID. These were chosen because there are a large number of already existing mappings of these identifiers. BioMart [24] was relied upon to create the mappings because of the amount and quality of information found in the databases available at the BioMart website (www.biomart.org). The data were output into a highly configurable format that was input into our local database. The Universal Translator program used data from the Uniprot website as well as the four interaction databases to create a mappings database between Uniprot, Entrez Gene, and Ensembl IDs. This database allows for the Interactome Merger application to operate much quicker.

Finally, an Interactome Merger application (source code also in Additional file 1) was developed to pull data from the four interaction databases and, using the database created by the Universal Translator and the mappings, make a merged interaction database. Interactome Merger was responsible for using the data in the Universal Translator database and mappings databases to convert interactions contained in DIP, BIND, BioGrid, and IntAct into a standardized format where each interaction had at least one of the three chosen identifiers associated to each interactor.

**Figure 1:**
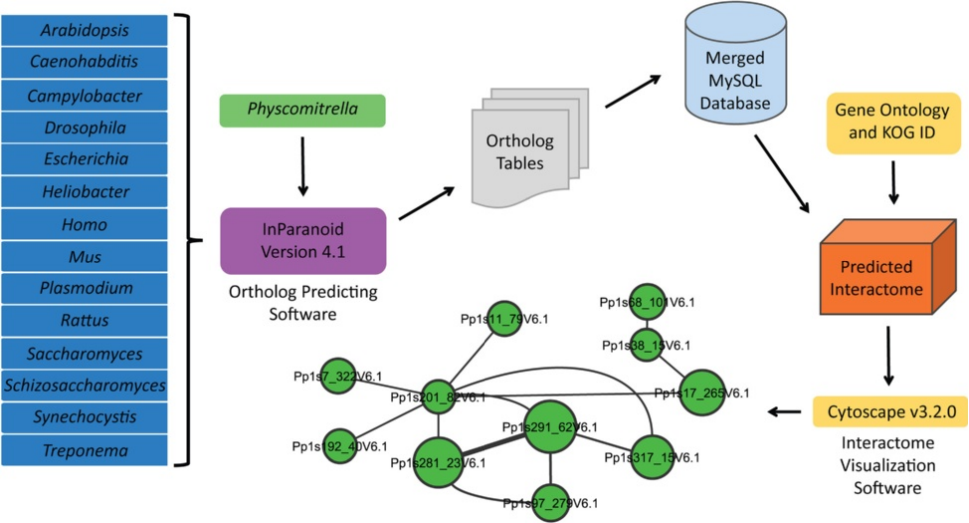
**A flow-chart can be used to visualize the process of generating the predicted interactome.** The predicted interactome of *Physcomitrella patens* was derived from orthologs of *Arabidopsis*, nematode, fruitfly, bacteria, mouse, rat, human and yeast using the InParanoid algorithm (See Methods). One to one orthology was used to query a MySQL database containing interaction data from BioGrid, BIND, DIP and Intact databases. A spreadsheet of orthologous interactions and their supporting information was generated and input into Cytoscape for the predicted interactome visualization.

The reference database was queried for interactions for which moss orthologs existed for both interacting partners. Output included source interactions, referenced metadata including placeholder ID and moss predicted interactions. This data spreadsheet was sorted for unique interactions and contains predicted orthologous protein IDs, type of experiment, organism the interaction was found, source database the interaction data were retrieved, and the PubMed ID for each interaction (Additional file 2: Table S1). The general process for assembling this database can be outlined visually using a flow-chart (Figure 1).

### Construction of predicted moss interactome

Interologs were determined using InParanoid ortholog predicting algorithm that compares, in pairwise manner, protein sequences derived from the gene models of *Physcomitrella patens* genome assembly V1.2 and annotation V1.6 from COSMOSS [19] against the gene models of *E. coli*, yeast, nematode worm, fruit fly, rat, mouse, and human [25,26]. The program separated outparalogs and orthologs into different clusters. These orthologs were loaded into a MySQL database that housed interactome datasets from BIND, BIOGRID, DIP, and IntAct databases [20-23]. One pair of proteins (moss, reference species) from each outparalog and ortholog cluster was selected to form the ortholog/outparalog one to one match (herein referred to as ortholog for brevity). A complete table of orthologs for all reference species is included as Additional file 3: Table S2.

The raw interolog data, unique interactions and calculation matrix for confidence value was maintained as a spreadsheet (Additional file 2: Table S1). This file contains 104,392 raw predicted interactions for *Physcomitrella*, reference species, type of methodology, and PubMed ID for the reference interaction. Moss protein IDs were provided for COSMOSS V1.2 and V1.6 annotation [19,27]. Current Uniprot, Genbank and other IDs (where available) can be obtained through Genonaut (https://www.cosmoss.org/annotation/genonaut). Duplicate interactions were removed to produce a dataset of 67,740 predicted interactions with the number of interactions for each protein, this constituted the unique moss interactome.

The confidence value (CV) for each unique interaction was calculated in the same way as Geisler-Lee et al. [11]. Essentially the CV is the total number of times the interaction is predicted from different experimental references, with a multiplier added for different experimental methods, and different reference species. Thus the CV of an interaction with evidence from 4 different references using 2 different experimental methods and 3 different species would be 4 × 2 × 3 = 24, while one with 4 references all using the same experimental method in the same species would be 4 × 1 × 1 = 4. This metric thus favours different experimental methodology and evolutionary conservation of the interaction.

Added to this spreadsheet were the KOG descriptions and definitions for each protein, which comprise the node attributes for the moss interactome. These data were loaded into Cytoscape 3.0.2 [28,29] and the predicted protein-protein interactome was constructed and displayed in the organic layout, thus rendering the ball and stick interactome model.

### Interactome analysis

Analysis of the overrepresented GO categories was conducted using the BiNGO v2.41 [30] plugin available through the Cytoscape web interface. The following settings were used to generate the network visualization; get the cluster from network and visualize overrepresented clusters after correction, perform hypergeometric test against the whole annotation with the Benjamini & Hochberg False Discovery Rate correction at the 0.05 significance level. Since the main concern was with the categories associated with plants, the GO Slim Plant ontology file was selected for cross-referencing against our custom Moss GO Annotation file (Additional file 4: Table S3). The same settings were employed to determine the underrepresented clusters. Hub distribution analysis and comparison used the Arabidopsis interactome from Geisler-Lee et al. [11] and the human and yeast interactomes downloaded from BioGrid [23].

## Results

### Identification of moss gene orthologs

Protein sequences from whole genomes of reference species were compared to those in the genome of *Physcomitrella patens* in a pairwise fashion using reciprocal BLASTs to separate inparalogs from orthologs and outparalogs with the InParanoid software package [25,26,31]. Of the nearly 28,000 genes in moss, only a few had true orthologs in yeast and animal reference species (Additional file 3: Table S2). The majority of orthologs present in the predicted interactome were from animal or fungus. Where multiple possible orthologs occurred, only one was selected for strict one-to-one orthology. The inparalogs were deliberately left out of the interaction prediction. The reasoning is that the last common species ancestor for most reference genomes is very early in the evolution of eukaryotes, thus each lineage has had enormous amount of time to diverge. Where such gene duplication has occurred, the resulting lack of selection pressure was thought likely lead to strong protein/gene neofunctionalization or subfunctionalization (or gene loss). Given the long evolutionary time, it was considered unlikely that a completely redundant function will remain, and thus cannot expect that such divergent inparalogs would have all the same interacting partners. Each moss gene was thus partnered with a single ortholog from each of the reference species where they occurred; this was assembled in MySQL and constituted the orthology database.

### Assembly of the reference database and interolog prediction

**Figure 2:**
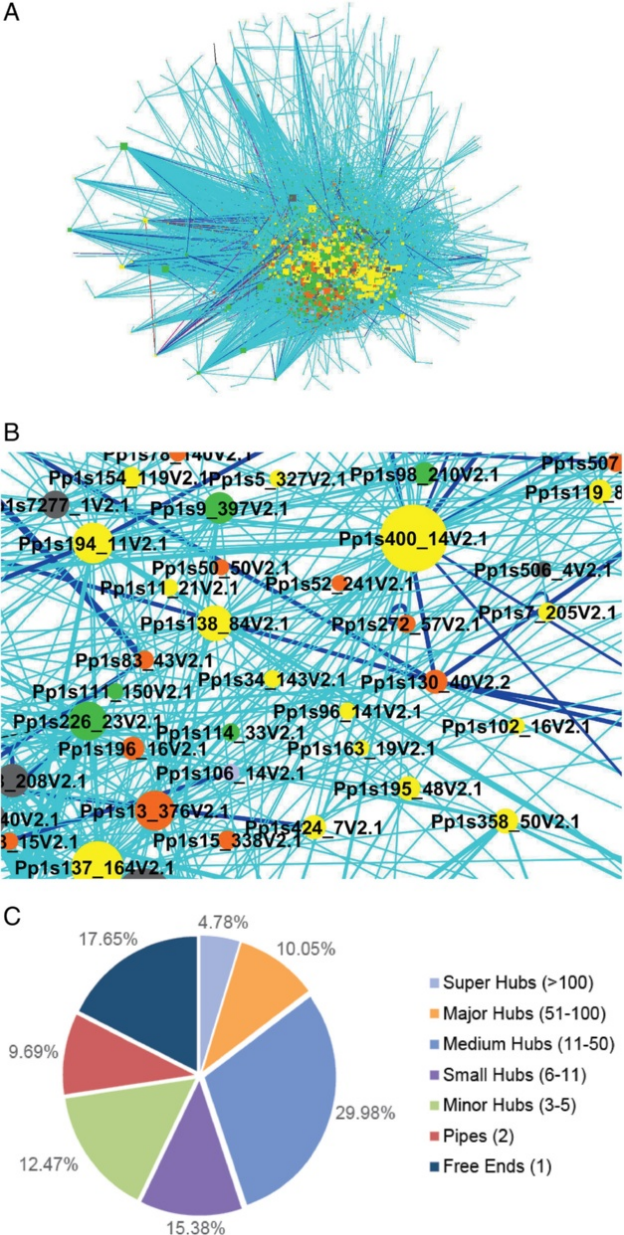
**The** ***Physcomitrella patens*** **network was viewed in Cytoscape and the hub distribution of proteins in the network were analysed. (A)** Large hairy ball of the 67,740 non-redundant interactions in the organic view of Cytoscape. **(B)** Detailed view showing layers of interacting proteins. **(C)** Pie chart of hub distribution by node class; “Free ends” are defined as have a single interaction, “pipes” have two interactions, and grouped hubs based on numbers of interactions. Numbers in parentheses indicate number of interacting proteins used to determine hub size.

More than 104,000 total interactions were predicted from different references including 67,740 unique interactions from 5,694 different *Physcomitrella patens* proteins (Additional file 2: Table S1). When visualized in its entirety in Cytoscape v3.2.0, the interactome looks like a ball of densely tangled circles and lines (Figure 2a,b). Like a map of roads and towns, this interactome is most useful when plotting connections between genes or pathways of interest and their neighbours, which is made possible in Cytoscape by the select node and zoom tools. Individual interactions were systematically evaluated using an evidence-based confidence value to assess the quality of the predicted interactions. 3936 high confidence (CV > 10), 10,318 medium confidence (CV between 2 and 10), and 53,400 low confidence (CV = 1) interactions were found in moss. The prediction efficiency of different confidence levels was evaluated by comparing the current experimentally determined interactions accumulated at the BioArray Resource [32] to the predicted interactions using the same interlog method and CV calculation for Arabidopsis by Geisler-Lee et al. [11]. The BAR resource combined the work of several high throughput experimental studies [8,33] with individual experiments culled from over 1190 publications (full list available at http://bar.utoronto.ca/interactions/cgi-bin/arabidopsis_interactions_viewer.cgi). The 37645 experimentally determined interactions in Arabidopsis were compared to the 72266 predicted interactions in Arabidopsis from non-plant reference genomes, and 1450 matched. The expected overlap between these datasets by chance alone (equal number of random protein pairs) was 91. This did not account for non-overlapping protein sets (proteins present in one set but absent in the other), nor did it account for the non-overlapping bias, as experimental interactomes tend to be biased towards plant specific genes, of which none would have occurred in the Arabidopsis predicted interactome. When broken down by confidence value, there was only slight improvement comparing interactions with low CV to those with medium CV, and there was 2.1 fold enrichment when comparing interactions with low and high confidence values.

**Figure 3:**
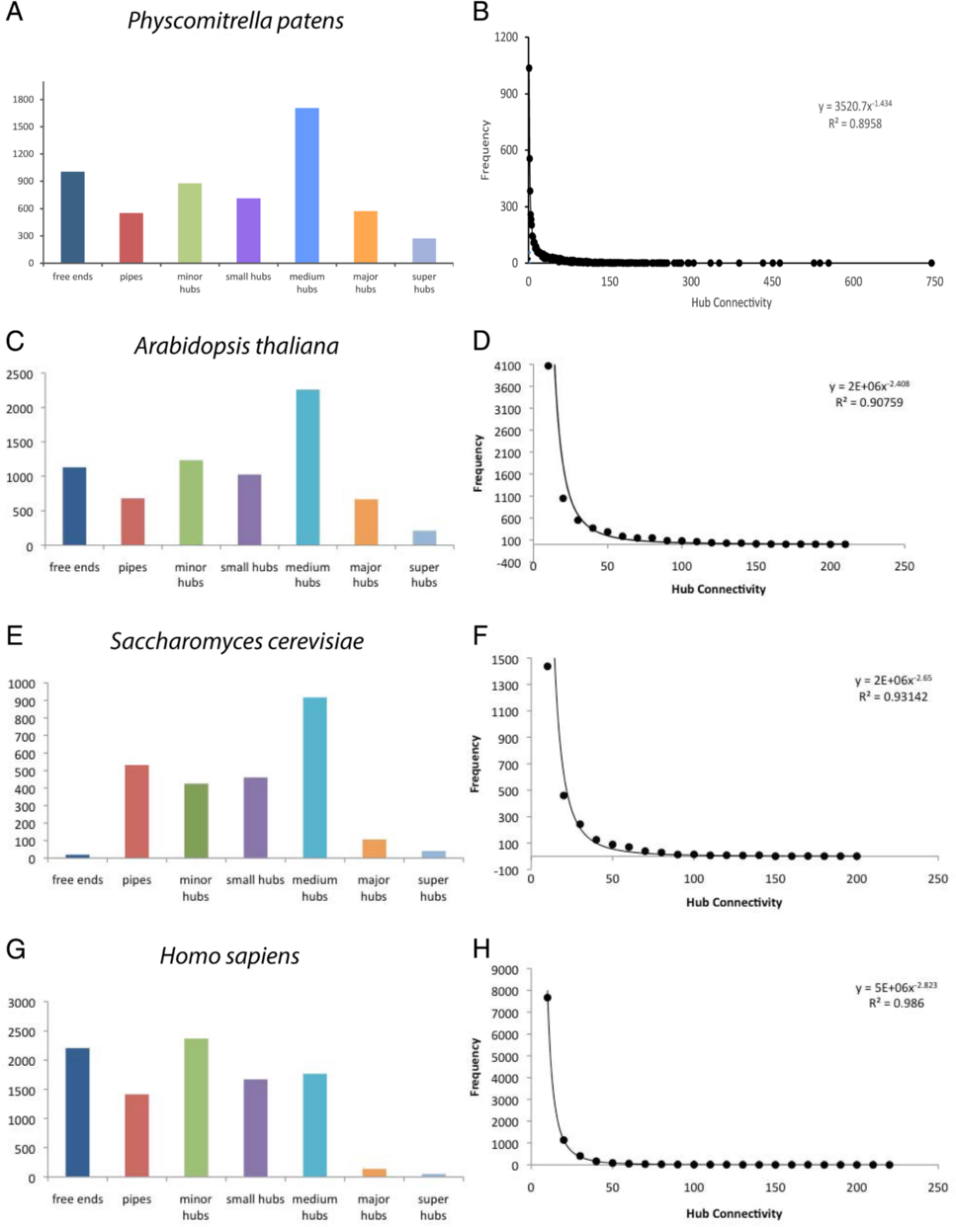
**Frequency distributions of hub sizes among** ***Physcomitrella patens*** **,** ***Arabidopsis thaliana*** **,** ***Saccharomyces cerevisiae*** **, and** ***Homo sapiens*** **.** Graphs for the frequency distribution of hub (protein) connectivity in shown both as a bar chart by node classes (left) and binned every 10 connections (right) in **A**, **B**: *Physcomitrella patens* (predicted, this work), **C**, **D**: *Arabidopsis thaliana* (predicted by [11]), **E**, **F**: *Saccharomyces cerevisiae* (experimentally determined; from BioGrid), and **G**, **H**: *Homo sapiens* (experimentally determined; from BioGrid).

The topology of the whole interactome was evaluated by dividing it into groups of proteins based on the number interactions (connectivity in Figure 2c). Greater than half the proteins are major hubs with between 51 and 100 interactions. In comparison, hub distributions in yeast and Arabidopsis are primarily medium in size between 11 and 50 interactions while the human protein interactome has more minor hubs (Figure 3a,c,e,g). Regardless of hub distributions in the interactomes of moss, Arabidopsis, yeast, and human, protein connectivity follows a scale free power law distribution (Figure 3b,d,f,h).

**Figure 4:**
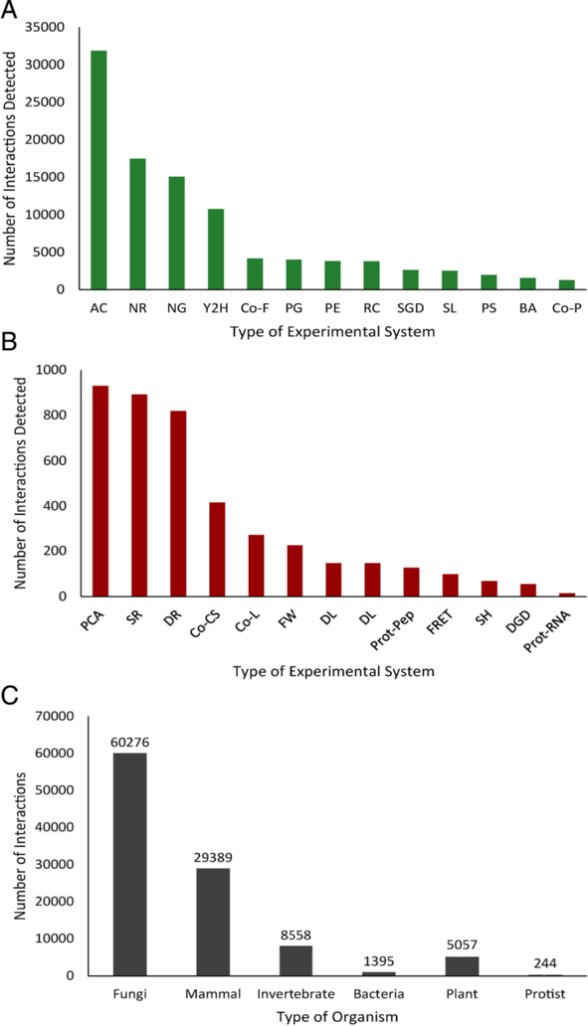
**A frequency distribution of interactions by experiment type and organism was analyzed.** Histogram showing numbers of interactions by type of experimental system**.** Major contributions shown in **(A)** and a smaller scale of contributions shown in **(B)**. Code abbreviations are as follows: NR, Not reported; AC, Affinity Capture; NG, Negative Genetic; Y2H, Yeast-two-hybrid; PE, Phenotypic Enhancement; SL, Synthetic Lethality; SGD, Synthetic Growth Defect; DR, Dosage Rescue; PS, Phenotypic Suppression; PG, Positive Genetic; RC, Reconstituted Complex; Co-P, Co-purification; SR, Synthetic Rescue; BA, Biochemical Activity. PCA, Principal Components Analysis; Co-F, Co-fractionation; Co-CS, Co-crystal Structure; Co-L, Co-localization; DL, Dosage Lethality; FW, Far Western; SH, Synthetic Haploinsufficiency; DGD, Dosage Growth Defect; Prot, Protein/Peptide; FRET. **(C)** Histogram of interactions by organism type.

Large hub sizes for proteins in both predicted and experimentally determined interactomes may be attributed to several factors. The most common high throughput experimental techniques used in reference organisms included Affinity Capture and Yeast Two Hybrid (Figure 4a,b), which can generate experimental false positive results [34]. This prompted the use of an experiment multiplier (E) when calculating confidence values. Additionally, conserved proteins tend to have more interacting partners and tend to be more essential [35], and this might skew the structure of the network composed of mostly conserved proteins towards mid to large hub sizes. Finally, it might indeed be true that this many potential interactions are possible, but that only a subset of these interactions actually occur in any given living cell or tissue due to differential expression of genes encoding the proteins. The distribution of species investigated was determined by counting the number experiments for each species, most coming from fungi (yeast) or animal references (Figure 4c). Approximately 5,130 moss predicted interactions that come from plant and cyanobacterial reference organisms (i.e. Arabidopsis and *Synechocystis*) were detected. Many of these interactions are highly conserved across eukaryotes (Additional file 5: Table S4), making evolutionary comparisons of plant networks feasible.

### Highly-connected nodes and conserved eukaryotic orthologs

**Figure 5:**
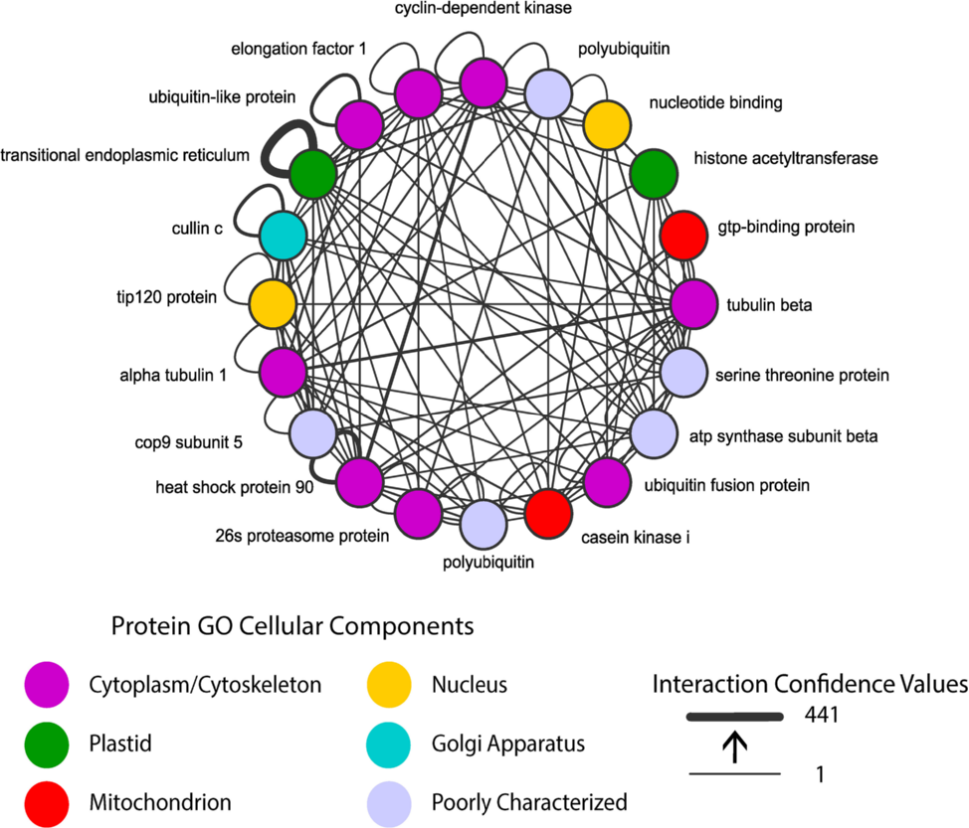
**The top twenty hubs in the** ***Physcomitrella*** **interactome were elucidated from the network.** The hubs represent the most highly connected proteins found within the dataset.

A search for the most connected proteins within the moss interactome revealed the largest hubs (greatest number of interacting partners) are linked to each other with high degrees of confidence (Figure 5). Many of these hubs are involved with the ribosome, nuclear DNA repair, ubiquitin, proteosome, and the cytoskeleton. These likely represent strongly conserved pathways that have not altered significantly during eukaryotic evolution. Heat shock proteins (HSP 90 and HSP 60) were also among the most highly connected proteins, possibly due to their ubiquitous involvement in forming dimers with numerous other proteins. An interesting node is Pp1s323_19V6.1, a GDP-mannose pyrophosphorylase enzyme that converts mannose to GDP-mannose used in cell wall biosynthesis, protein glycosylation and ascorbic acid biosynthesis. This protein appears to have many more predicted interacting partners (237; Additional file 6: Table S5) in moss than the ortholog for Arabidopsis CYT1/AT2G39770 (88 partners) [11,32]. Of the additional interacting partners in moss most came from references not used in the construction of the Arabidopsis predicted interactome, but 37 interacting partners had no Arabidopsis ortholog. These represent moss genes with no matching ortholog in Arabidopsis, but with orthologs other reference species (i.e. yeast, human, *E. coli*).

The moss interactome, like other partially completed interactomes for Arabidopsis, human, drosophila and rice is surprisingly well connected, with most proteins assembled into a single interacting ball. One early theory for the possible global organisation of biological pathways is that of densely connected subnetworks that are only connected to each other due to the presence of highly connected hubs such as HSPs; this is referred to as the “party and date” hub model. A second model, observed in yeast, is that there is dense interconnection throughout the entire proteome and few or no separate subnetworks connected by date hubs; this is known as the stratus (cloud) model [36-39]. The robustness of the interactome was tested by deleting the top 20 connected proteins to determine if the moss interactome core fits the party-date model or the stratus model. The deletion of these proteins had no overall effect on the interactome topology. All remaining proteins stayed connected and the distribution of hub size was not affected significantly except for the top twenty.

The top one hundred most conserved interactions were examined across reference eukaryotes. Like the most conserved hubs, the conserved interactions were found to represent core biological processes such as DNA transcription and polymerization, RNA polymerization, cell cycle, and vesicle associated interactions (Additional file 5: Table S4). However, within this list of fifty interactions there are ten proteins with completely unknown functions and among those, two of the unknown proteins interact with each other. This occurs due to the high throughput experimental methodologies tend to investigate interactions without knowing what the proteins do (i.e. somewhat double blind). Thus there are still core-conserved processes that are not understood thus proteins involved in these are good candidates for future research of significance to all of biological science.

### Analysis of gene ontology

**Figure 6:**
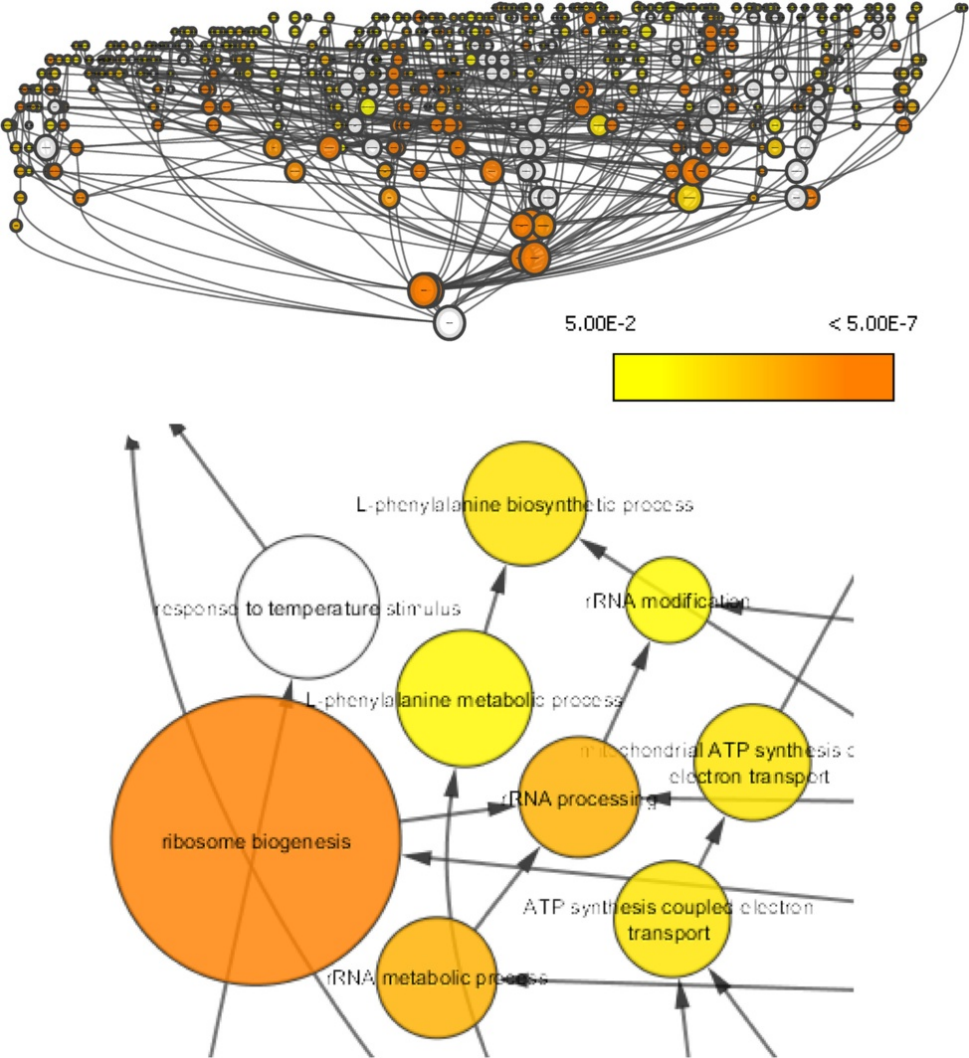
**A BiNGO analysis of the** ***Physcomitrella patens*** **predicted interactome was performed in order to determine over-represented cellular processes.** Node size is related to the number of times a protein in that category is found in the predicted interactome. Node color indicates level of significance (i.e. p-value) at the 0.05 level. (See Additional file 5: Table S4 for complete protein list and their p-values).

The conservation of proteins using the interlog method will potentially bias the resulting network towards conserved processes. To learn what these conserved processes were, genes in the interactome were annotated by Gene Ontology, using a reference list of approximately 17,000 GO annotations for *Physcomitrella patens* [19]. An analysis for enriched and depleted processes revealed that proteins involved in intracellular and cytoplasmic metabolic and catalytic processes are overrepresented likely due to their conserved nature. Additionally the protein binding category was enriched due to physical interaction requirement for inclusion in the interactome (Figure 6, Additional file 7: Table S6). Within the cellular and metabolic processes, a majority of overrepresented proteins involved DNA and protein metabolism, likely due to these processes being conserved in all life and consequently being the most studied in the reference organisms. Also cellular component organization and biogenesis are overrepresented in our interactome. There were relatively few, but significant, representation of proteins involved in cell cycle, growth, embryonic development, cell differentiation, and behavior. The catalytic and binding activities most represented in the interactome relate to hydrolase and transferase enzymes, as well as nucleic acid, protein, and nucleotide binding. A complete protein list from the analysis is provided in Additional file 5: Table S4.

### Orthology and comparison of moss and Arabidopsis genomes

*Physcomitrella* appears to have more orthologs in general, although this value does not represent a significant increase. For example: moss has 2,999 orthologous proteins to human and 1,793 orthologous proteins to yeast, while Arabidopsis has 2,708 and 1,708 proteins respectively. Of the 2,999 human orthologs moss and Arabidopsis share 1,485 proteins, but moss has 896 proteins with unique orthology to human proteins. Most interesting though, is the orthology of arachidonate 5-lipoxygenase (5-LOX) in moss and human. This enzyme in humans adds oxygen to fatty acids for form arachidonic acids (animal fat) that are implicated in prostate tumor growth and asthma [40]. Arachidonic acids are significantly less abundant in angiosperms with respect to linoleic and α-linoleic acids, while the inverse is the condition in algae and bryophytes [41]. However arachidonate 12-lipoxygenases (12-LOX), not 5-LOX, significantly prefer arachidonic acid as a substrate. Therefore we speculate that the 5-LOX enzyme activity is reduced due to purifying selection for 12-LOX in *Physcomitrella patens*. This discovery highlights that mosses and other bryophytes may possess enzymes and pathways that are shared with non-plant eukaryotes, but are reduced or absent in angiosperms or vascular plants. As each sister lineage of land plants diverged from their last common ancestor, they may have selectively lost different conserved pathways. These differences are detected by ortholog and interolog analysis at the whole genome level.

## Discussion

### Topology and features of the predicted interactome

A predicted protein interactome for *Physcomitrella patens* was constructed using the interolog method based on orthologous genes across 14 reference organisms. Nearly 68,000 unique interactions were recovered from 5,695 proteins in *Physcomitrella patens*. The increase in the number of reference species more than doubled the number of interactions, and added just over 400 more proteins than were found in Arabidopsis using the same methods [11]. Increased taxon sampling resulted in increased CV for the most highly conserved interactions. Interactions with the highest CV are typically those involving proteins from highly conserved processes such as DNA replication and polymerization, RNA polymerization, endomembrane trafficking, metabolism and mitosis. Consequently accurate prediction of protein interaction pathways is possible for these processes in moss. Additionally proteins involved in formation of the proteosome complexes are overrepresented in the moss interactome. This both points out the conserved nature of this complex and illustrates the limitation of this methodology. As more interactomic data become available from reference species, there is greater confidence in the predicted proteins involved in deeply conserved processes [1,2,6,7,9,11,13].

Functional annotation of most (>99%) proteins in genome databases is sequence homology based without any experimental data. This work adds to this annotation by inference based on the presence of known interacting partners. When two unknown proteins interact with each other such inferences are not possible, however the co-occurrence of these proteins across several taxa makes it interesting because whole processes, completely unknown as to their function, can be conserved across eukaryotes. This implies that there are still fundamental biological pathways as yet unexplored. The top hubs (i.e. proteins most interacting partners) represent well-known fundamental biological processes (Figure 5). Given their importance to the interactome one would expect removal of these proteins would lead to a breakdown into several disconnected networks. Yet removal of these highly connected nodes did not result in such a breakdown. These results suggest the moss predicted protein interactome fits a stratus structure of connectivity [36]. This type of network is composed of densely connected hubs that have high degrees of overlap of interacting proteins. This differs from the view that protein interaction networks exhibit a party/date hub or altocumulus structure where subnetworks interact through a very few (date) hubs [39]. Protein interactomes that fit a stratus structure rather than an altocumulus structure support data demonstrating that many proteins have multiple functions or participate in multiple complexes [37,38]. However high density connectedness found in stratus-like interactomes are less amenable to finding specific modules within the structure [36].

When the general features were looked at for the moss, Arabidopsis, yeast and human interactomes a key difference was discovered in the distribution of hubs. *Physcomitrella patens* has more major hubs (51–100 interactions) while yeast and human have more minor, small, and medium hubs. The Arabidopsis interactome connectivity was more similar to the moss genome than it was to either yeast or human. Moss has fewer free-ends and pipes when compared to Arabidopsis, but this could due to the fewer number of reference species used to generate the Arabidopsis dataset [11]. Since hub size is directly associated with numbers of interacting proteins, the increased number of reference species increases the number of predicted interacting proteins. The increased number of interactions would result in a shift in the types of hubs represented in the moss interactome. Regardless of this shift, hub connectivity follows a scale-free power law distribution (Figure 3), a feature found in other interactomes, metabolic networks, and neural networks [42].

Another feature of *Physcomitrella patens* is that it is a paleopolyploid as result from an ancient genome duplication that preferentially maintained a large complement of metabolic genes [27,43] encoding for proteins that are overrepresented in the predicted interactome. While not significantly different, in general moss appears to have more orthologs with the reference species than does Arabidopsis. This is expected given that the genomes of *Physcomitrella patens* and Arabidopsis have very similar numbers of protein coding sequences. However the common ancestor to the Brassicales also underwent a genome duplication event followed by subsequent genome reductions in Arabidopsis resulting in biased retention toward transcriptional regulation and signal transduction [44].

### “Guilt-by-association” and analysis of subnetworks

The utility of predicted interactomes lies in the “guilt-by-association” model of predicting proteins in a pathway under the assumption that orthologous proteins have similar functions [2,4,11,45]. Roadmaps are provided for all the conserved pathways in moss and assign pathway position and function through interactions. This was demonstrated, with high confidence, in predicting disease genes in humans [46,47].

**Figure 7:**
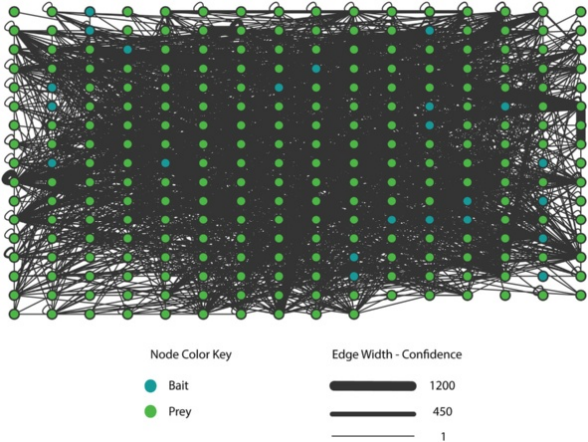
**A bait and prey method was used to reconstruct a sub-network using the** ***Physcomitrella*** **predicted interactome.** Two proteins known to be involved in the Calvin-Benson-Bassham cycle were used as bait (blue color nodes) and their first neighbors are the captured prey (green color nodes). These two known CBB-cycle proteins do not directly interact, but interact with several other proteins that serve as intermediates. Additional file 8: Table S7 contains the complete protein list and annotations used for this analysis.

This *Physcomitrella* interactome can be used to analyze specific subnetworks and further characterize key interacting proteins. Using the interactome, one can view interactions between proteins that are known to be involved in a certain processes (bait) and others that directly interact with the bait (prey). Of all known CBB-associated proteins, 23 were found to exist in the dataset and were chosen as bait resulting in a prey capture 242 proteins (Figure 7, Additional file 8: Table S7). Finding key prey proteins can be accomplished by look at the ratio of that protein’s connectivity within the subnetwork to its overall connectivity. Prey proteins that exclusively occur in this subnetwork include a carbohydrate transmembrane transporter (Pp1s88_88V6.1), isocitrate dehydrogenase (Pp1s78_143V6.1), glycosyltransferase (Pp1s90_132V6.1), UDP-acetylglucosamine acyltransferase (Pp1s377_21V6.1), and a protein with a domain of unknown function (Pp1s30_41V6.1). The average connectivity ratio in this subnetwork was 0.33 suggesting that many of these proteins are specific to this process. Reconstruction of subnetworks is aided through this “bait and prey” model and can help elucidate mechanisms that emerge from the network’s activity.

## Conclusion

We developed the moss interactome to increase the community resource base by improving the annotation of moss proteins by adding predicted interactions and allowing guilt-by-association functional annotation. The ability to view an interactome in its entirety facilitates the verification of known interacting proteins and discovery of new proteins involved in a particular pathway or process. The availability of an annotated interactome for the moss provides an invaluable resource and focal point for system-scale analysis of this bryophyte model organism. The addition of moss, a plant representative of about 450 million years divergent evolution from seed plants like Arabidopsis, to interactomic research greatly expands the possibility of conducting comparative analyses thus giving additional insight into network evolution of land plants.

## Additional files

Additional file 1:
**Software package used in generating the interactome from databases.**
Additional file 2: Table S1. Moss Interolog Database. Additional file 3: Table S2. Moss Ortholog Database. Additional file 4: Table S3. Moss GO Annotation. Additional file 5: Table S4. List of interactions conserved in at least 5 eukaryotes. Additional file 6: Table S5. GDP-Mannose PPase interaction comparison. Additional file 7: Table S6. BinGO Analysis. Additional file 8: Table S7. CBB Subnetwork.

## Abbreviations

PPI: Protein-protein interaction
LOX: Lipoxygenasae
HSP: Heat-shock protein
CBB: Calvin-Benson-Bassham cycle
