# Supplementary material for: Predicted protein-protein interactions in the moss Physcomitrella patens: a new bioinformatic resource
Source: BMC Bioinformatics. 2015 Mar 16;16(1):89. doi: 10.1186/s12859-015-0524-1 (PMC4384322; doi:10.1186/s12859-015-0524-1)
Supplement: Additional file 1: — Software package used in generating the interactome from databases. [file 12859_2015_524_MOESM1_ESM.zip › MySQL_Importer_v1/README.pdf]

How To Use MySQL Data Import

V1

Aaron Corley

Running the Program

To run the MySQL Data Import Program, simply double-click on MySQL_DataImport.jar. If you are having troubles getting this to work, please ensure that you have the latest JRE installed. The latest JRE can be found and downloaded here.

Format of the Input File

The input file must have the following format in order for this program to produce the expected output(note that the Line identifiers are merely used to represent where specific information should be in the file):

For comma-separated files:

```
Line 1: FieldName1,FieldName2,…
Line 2: Field1Value, Field2Value,…
…
```

For tab-separated files:

```
Line 1: FieldName1   FieldName2     …
Line 2: Field1Value Field2Value     …
…
```

Format of the Output File

This program creates an output file with the following format:

```
INSERT INTO INPUT_FILE_NAME VALUES('Field1Value','Field2Value',…);
…
```

Where `INPUT_FILE_NAME` represents the name of the file that you are using for input.

Other Notes

- This program does not generated a CREATE statement. In other words, a database/table with name `INPUT_FILE_NAME` should already exist before you try to run the script that this program generates.

- This program generates a script that treats each FieldValue as a string; therefore, ensure that the fields in the destination database are set to VARCHAR or CHAR of appropriate length.
