# Supplementary material for: Predicted protein-protein interactions in the moss Physcomitrella patens: a new bioinformatic resource
Source: BMC Bioinformatics. 2015 Mar 16;16(1):89. doi: 10.1186/s12859-015-0524-1 (PMC4384322; doi:10.1186/s12859-015-0524-1)
Supplement: Additional file 1: — Software package used in generating the interactome from databases. [file 12859_2015_524_MOESM1_ESM.zip › MySQL_Importer_v1/javadoc/serialized-form.html]

Serialized Form


---


|  |  |  |  |  |  |  |  |  |  |
| --- | --- | --- | --- | --- | --- | --- | --- | --- | --- |
| |  |  |  |  |  |  |  | | --- | --- | --- | --- | --- | --- | --- | | **Package** | Class | Use | **Tree** | **Deprecated** | **Index** | **Help** | | |  |
| PREV   NEXT | **FRAMES**    **NO FRAMES**     **All Classes** |


---


# Serialized Form


---

| **Package** **Source** |
| --- |

| **Class Source.DataImportGUI extends javax.swing.JFrame implements Serializable** | |
| --- | --- |

| **Serialized Fields** |
| --- |

### inFile

```
java.io.File inFile
```

---

### outFile

```
java.io.File outFile
```

---

### di

```
DataImport di
```

---

### csvRadioButton

```
javax.swing.JRadioButton csvRadioButton
```

---

### exitButton

```
javax.swing.JButton exitButton
```

---

### fileExit

```
javax.swing.JMenuItem fileExit
```

---

### fileMenu

```
javax.swing.JMenu fileMenu
```

---

### getInputFileButton

```
javax.swing.JButton getInputFileButton
```

---

### getOutputFileButton

```
javax.swing.JButton getOutputFileButton
```

---

### inputFilePath

```
javax.swing.JTextField inputFilePath
```

---

### inputFormatGroup

```
javax.swing.ButtonGroup inputFormatGroup
```

---

### jLabel1

```
javax.swing.JLabel jLabel1
```

---

### jLabel2

```
javax.swing.JLabel jLabel2
```

---

### jMenuBar1

```
javax.swing.JMenuBar jMenuBar1
```

---

### outputFilePath

```
javax.swing.JTextField outputFilePath
```

---

### progressBar

```
javax.swing.JProgressBar progressBar
```

---

### runButton

```
javax.swing.JButton runButton
```

---

### tsvRadioButton

```
javax.swing.JRadioButton tsvRadioButton
```

---


|  |  |  |  |  |  |  |  |  |  |
| --- | --- | --- | --- | --- | --- | --- | --- | --- | --- |
| |  |  |  |  |  |  |  | | --- | --- | --- | --- | --- | --- | --- | | **Package** | Class | Use | **Tree** | **Deprecated** | **Index** | **Help** | | |  |
| PREV   NEXT | **FRAMES**    **NO FRAMES**     **All Classes** |


---
