# Supplementary material for: Predicted protein-protein interactions in the moss Physcomitrella patens: a new bioinformatic resource
Source: BMC Bioinformatics. 2015 Mar 16;16(1):89. doi: 10.1186/s12859-015-0524-1 (PMC4384322; doi:10.1186/s12859-015-0524-1)
Supplement: Additional file 1: — Software package used in generating the interactome from databases. [file 12859_2015_524_MOESM1_ESM.zip › MySQL_Importer_v1/javadoc/index.html]

Generated Documentation (Untitled)


<H2>
Frame Alert</H2>
<P>
This document is designed to be viewed using the frames feature. If you see this message, you are using a non-frame-capable web client.
<BR>
Link to<A HREF="Source/package-summary.html">Non-frame version.</A>
