# Supplementary material for: Predicted protein-protein interactions in the moss Physcomitrella patens: a new bioinformatic resource
Source: BMC Bioinformatics. 2015 Mar 16;16(1):89. doi: 10.1186/s12859-015-0524-1 (PMC4384322; doi:10.1186/s12859-015-0524-1)
Supplement: Additional file 1: — Software package used in generating the interactome from databases. [file 12859_2015_524_MOESM1_ESM.zip › MySQL_Importer_v1/javadoc/constant-values.html]

Constant Field Values


---


|  |  |  |  |  |  |  |  |  |  |
| --- | --- | --- | --- | --- | --- | --- | --- | --- | --- |
| |  |  |  |  |  |  |  | | --- | --- | --- | --- | --- | --- | --- | | **Package** | Class | Use | **Tree** | **Deprecated** | **Index** | **Help** | | |  |
| PREV   NEXT | **FRAMES**    **NO FRAMES**     **All Classes** |


---


# Constant Field Values


---

**Contents**

- Source.\*

| Source.\* |
| --- |

| Source.DataImport | | |
| --- | --- | --- |
| `public static final int` | `CSV` | `1` |
| `public static final int` | `TSV` | `2` |

| Source.FileReader | | |
| --- | --- | --- |
| `public static final java.lang.String` | `COMMA_DELIMITED` | `"\\,"` |
| `public static final java.lang.String` | `SPACE_DELIMITED` | `"\\s"` |
| `public static final java.lang.String` | `TAB_DELIMITED` | `"\t"` |

---


|  |  |  |  |  |  |  |  |  |  |
| --- | --- | --- | --- | --- | --- | --- | --- | --- | --- |
| |  |  |  |  |  |  |  | | --- | --- | --- | --- | --- | --- | --- | | **Package** | Class | Use | **Tree** | **Deprecated** | **Index** | **Help** | | |  |
| PREV   NEXT | **FRAMES**    **NO FRAMES**     **All Classes** |


---
