# Supplementary material for: Predicted protein-protein interactions in the moss Physcomitrella patens: a new bioinformatic resource
Source: BMC Bioinformatics. 2015 Mar 16;16(1):89. doi: 10.1186/s12859-015-0524-1 (PMC4384322; doi:10.1186/s12859-015-0524-1)
Supplement: Additional file 1: — Software package used in generating the interactome from databases. [file 12859_2015_524_MOESM1_ESM.zip › MySQL_Importer_v1/javadoc/index-files/index-5.html]

I-Index


---


|  |  |  |  |  |  |  |  |  |  |
| --- | --- | --- | --- | --- | --- | --- | --- | --- | --- |
| |  |  |  |  |  |  |  | | --- | --- | --- | --- | --- | --- | --- | | **Package** | Class | Use | **Tree** | **Deprecated** | **Index** | **Help** | | |  |
| **PREV LETTER**   **NEXT LETTER** | **FRAMES**    **NO FRAMES**     **All Classes** |


C D F G I M R S T U 

---


## **I**

**indexOf(String)** - Method in class Source.FileReader: This function is similar to String's 'indexOf' function, it searches for needle and returns the index of the first character in needle, and -1 if needle is null or the string can't be found in the file. **indexOf(String, int)** - Method in class Source.FileReader: This function is similar to String's 'indexOf' function, it searches for needle and returns the index of the first character in needle, and -1 if needle is null or the string can't be found in the file. **isReady()** - Method in class Source.FileReader: Returns whether or not the file is ready to be read from (usually: if the end of the file has been reached or not)

---


|  |  |  |  |  |  |  |  |  |  |
| --- | --- | --- | --- | --- | --- | --- | --- | --- | --- |
| |  |  |  |  |  |  |  | | --- | --- | --- | --- | --- | --- | --- | | **Package** | Class | Use | **Tree** | **Deprecated** | **Index** | **Help** | | |  |
| **PREV LETTER**   **NEXT LETTER** | **FRAMES**    **NO FRAMES**     **All Classes** |


C D F G I M R S T U 

---
