# Supplementary material for: Predicted protein-protein interactions in the moss Physcomitrella patens: a new bioinformatic resource
Source: BMC Bioinformatics. 2015 Mar 16;16(1):89. doi: 10.1186/s12859-015-0524-1 (PMC4384322; doi:10.1186/s12859-015-0524-1)
Supplement: Additional file 1: — Software package used in generating the interactome from databases. [file 12859_2015_524_MOESM1_ESM.zip › MySQL_Importer_v1/javadoc/index-files/index-4.html]

G-Index


---


|  |  |  |  |  |  |  |  |  |  |
| --- | --- | --- | --- | --- | --- | --- | --- | --- | --- |
| |  |  |  |  |  |  |  | | --- | --- | --- | --- | --- | --- | --- | | **Package** | Class | Use | **Tree** | **Deprecated** | **Index** | **Help** | | |  |
| **PREV LETTER**   **NEXT LETTER** | **FRAMES**    **NO FRAMES**     **All Classes** |


C D F G I M R S T U 

---


## **G**

**getCurrentLine()** - Method in class Source.FileReader: This method returns the line that FileReader is currently on. **getNextLine()** - Method in class Source.FileReader: This function returns the next line in the file. **getNextWord()** - Method in class Source.FileReader: This function returns the next word in the line that is currently being read. **getNumberOfLines()** - Method in class Source.FileReader: Returns the number of lines in the file **getNumberOfWords()** - Method in class Source.FileReader: Returns the number of words in the current line

---


|  |  |  |  |  |  |  |  |  |  |
| --- | --- | --- | --- | --- | --- | --- | --- | --- | --- |
| |  |  |  |  |  |  |  | | --- | --- | --- | --- | --- | --- | --- | | **Package** | Class | Use | **Tree** | **Deprecated** | **Index** | **Help** | | |  |
| **PREV LETTER**   **NEXT LETTER** | **FRAMES**    **NO FRAMES**     **All Classes** |


C D F G I M R S T U 

---
