# Supplementary material for: Predicted protein-protein interactions in the moss Physcomitrella patens: a new bioinformatic resource
Source: BMC Bioinformatics. 2015 Mar 16;16(1):89. doi: 10.1186/s12859-015-0524-1 (PMC4384322; doi:10.1186/s12859-015-0524-1)
Supplement: Additional file 1: — Software package used in generating the interactome from databases. [file 12859_2015_524_MOESM1_ESM.zip › MySQL_Importer_v1/javadoc/index-files/index-3.html]

F-Index


---


|  |  |  |  |  |  |  |  |  |  |
| --- | --- | --- | --- | --- | --- | --- | --- | --- | --- |
| |  |  |  |  |  |  |  | | --- | --- | --- | --- | --- | --- | --- | | **Package** | Class | Use | **Tree** | **Deprecated** | **Index** | **Help** | | |  |
| **PREV LETTER**   **NEXT LETTER** | **FRAMES**    **NO FRAMES**     **All Classes** |


C D F G I M R S T U 

---


## **F**

**FileReader** - Class in Source: This object is designed to simplify the process of reading from a file. **FileReader(String)** - Constructor for class Source.FileReader: This constructor accepts the path of the file to be opened as a string, opens it, and prepares the object to be used for reading. **FileReader(String, String)** - Constructor for class Source.FileReader: This constructor accepts the path of the file to be opened as a string, opens it, and prepares the object to be used for reading. **FileReader(File)** - Constructor for class Source.FileReader: This constructor accepts the path of the file to be opened as a File object, opens it, and prepares the object to be used for reading. **FileReader(File, String)** - Constructor for class Source.FileReader: This constructor accepts the path of the file to be opened as a File object, opens it, and prepares the object to be used for reading. **finalize()** - Method in class Source.FileReader: Closes the file when this object is destroyed.

---


|  |  |  |  |  |  |  |  |  |  |
| --- | --- | --- | --- | --- | --- | --- | --- | --- | --- |
| |  |  |  |  |  |  |  | | --- | --- | --- | --- | --- | --- | --- | | **Package** | Class | Use | **Tree** | **Deprecated** | **Index** | **Help** | | |  |
| **PREV LETTER**   **NEXT LETTER** | **FRAMES**    **NO FRAMES**     **All Classes** |


C D F G I M R S T U 

---
