# Supplementary material for: Predicted protein-protein interactions in the moss Physcomitrella patens: a new bioinformatic resource
Source: BMC Bioinformatics. 2015 Mar 16;16(1):89. doi: 10.1186/s12859-015-0524-1 (PMC4384322; doi:10.1186/s12859-015-0524-1)
Supplement: Additional file 1: — Software package used in generating the interactome from databases. [file 12859_2015_524_MOESM1_ESM.zip › MySQL_Importer_v1/javadoc/index-files/index-2.html]

D-Index


---


|  |  |  |  |  |  |  |  |  |  |
| --- | --- | --- | --- | --- | --- | --- | --- | --- | --- |
| |  |  |  |  |  |  |  | | --- | --- | --- | --- | --- | --- | --- | | **Package** | Class | Use | **Tree** | **Deprecated** | **Index** | **Help** | | |  |
| **PREV LETTER**   **NEXT LETTER** | **FRAMES**    **NO FRAMES**     **All Classes** |


C D F G I M R S T U 

---


## **D**

**DataImport** - Class in Source: This object will take data from a file (CSV or TSV), and create a MySQL script that will be able to import the data into a database. **DataImport(File, File, int, DataImportGUI)** - Constructor for class Source.DataImport: Creates a new DataImport thread **DataImportGUI** - Class in Source: This is the driver class for the MySQL DataImport program. **DataImportGUI()** - Constructor for class Source.DataImportGUI: Creates new form DataImportGUI

---


|  |  |  |  |  |  |  |  |  |  |
| --- | --- | --- | --- | --- | --- | --- | --- | --- | --- |
| |  |  |  |  |  |  |  | | --- | --- | --- | --- | --- | --- | --- | | **Package** | Class | Use | **Tree** | **Deprecated** | **Index** | **Help** | | |  |
| **PREV LETTER**   **NEXT LETTER** | **FRAMES**    **NO FRAMES**     **All Classes** |


C D F G I M R S T U 

---
