# Supplementary material for: Predicted protein-protein interactions in the moss Physcomitrella patens: a new bioinformatic resource
Source: BMC Bioinformatics. 2015 Mar 16;16(1):89. doi: 10.1186/s12859-015-0524-1 (PMC4384322; doi:10.1186/s12859-015-0524-1)
Supplement: Additional file 1: — Software package used in generating the interactome from databases. [file 12859_2015_524_MOESM1_ESM.zip › MySQL_Importer_v1/javadoc/Source/package-tree.html]

Source Class Hierarchy


---


|  |  |  |  |  |  |  |  |  |  |
| --- | --- | --- | --- | --- | --- | --- | --- | --- | --- |
| |  |  |  |  |  |  |  | | --- | --- | --- | --- | --- | --- | --- | | **Package** | Class | Use | **Tree** | **Deprecated** | **Index** | **Help** | | |  |
| PREV   NEXT | **FRAMES**    **NO FRAMES**     **All Classes** |


---


## Hierarchy For Package Source

## Class Hierarchy

- java.lang.Object
  - java.awt.Component (implements java.awt.image.ImageObserver, java.awt.MenuContainer, java.io.Serializable)
    - java.awt.Container
      - java.awt.Window (implements javax.accessibility.Accessible)
        - java.awt.Frame (implements java.awt.MenuContainer)
          - javax.swing.JFrame (implements javax.accessibility.Accessible, javax.swing.RootPaneContainer, javax.swing.WindowConstants)
            - Source.**DataImportGUI**- Source.**FileReader**- java.lang.Thread (implements java.lang.Runnable)
        - Source.**DataImport**

---


|  |  |  |  |  |  |  |  |  |  |
| --- | --- | --- | --- | --- | --- | --- | --- | --- | --- |
| |  |  |  |  |  |  |  | | --- | --- | --- | --- | --- | --- | --- | | **Package** | Class | Use | **Tree** | **Deprecated** | **Index** | **Help** | | |  |
| PREV   NEXT | **FRAMES**    **NO FRAMES**     **All Classes** |


---
