# Supplementary material for: Predicted protein-protein interactions in the moss Physcomitrella patens: a new bioinformatic resource
Source: BMC Bioinformatics. 2015 Mar 16;16(1):89. doi: 10.1186/s12859-015-0524-1 (PMC4384322; doi:10.1186/s12859-015-0524-1)
Supplement: Additional file 1: — Software package used in generating the interactome from databases. [file 12859_2015_524_MOESM1_ESM.zip › MySQL_Importer_v1/javadoc/Source/package-summary.html]

Source


---


|  |  |  |  |  |  |  |  |  |  |
| --- | --- | --- | --- | --- | --- | --- | --- | --- | --- |
| |  |  |  |  |  |  |  | | --- | --- | --- | --- | --- | --- | --- | | **Package** | Class | **Use** | **Tree** | **Deprecated** | **Index** | **Help** | | |  |
| PREV PACKAGE   NEXT PACKAGE | **FRAMES**    **NO FRAMES**     **All Classes** |


---

## Package Source

| **Class Summary** | |
| --- | --- |
| **DataImport** | This object will take data from a file (CSV or TSV), and create a MySQL script that will be able to import the data into a database. |
| **DataImportGUI** | This is the driver class for the MySQL DataImport program. |
| **FileReader** | This object is designed to simplify the process of reading from a file. |

---


|  |  |  |  |  |  |  |  |  |  |
| --- | --- | --- | --- | --- | --- | --- | --- | --- | --- |
| |  |  |  |  |  |  |  | | --- | --- | --- | --- | --- | --- | --- | | **Package** | Class | **Use** | **Tree** | **Deprecated** | **Index** | **Help** | | |  |
| PREV PACKAGE   NEXT PACKAGE | **FRAMES**    **NO FRAMES**     **All Classes** |


---
