# Supplementary material for: Predicted protein-protein interactions in the moss Physcomitrella patens: a new bioinformatic resource
Source: BMC Bioinformatics. 2015 Mar 16;16(1):89. doi: 10.1186/s12859-015-0524-1 (PMC4384322; doi:10.1186/s12859-015-0524-1)
Supplement: Additional file 1: — Software package used in generating the interactome from databases. [file 12859_2015_524_MOESM1_ESM.zip › MySQL_Importer_v1/javadoc/Source/FileReader.html]

FileReader


---


|  |  |  |  |  |  |  |  |  |  |
| --- | --- | --- | --- | --- | --- | --- | --- | --- | --- |
| |  |  |  |  |  |  |  | | --- | --- | --- | --- | --- | --- | --- | | **Package** | **Class** | **Use** | **Tree** | **Deprecated** | **Index** | **Help** | | |  |
| **PREV CLASS**   NEXT CLASS | **FRAMES**    **NO FRAMES**     **All Classes** |
| SUMMARY: NESTED | FIELD | CONSTR | METHOD | DETAIL: FIELD | CONSTR | METHOD |


---


## Source Class FileReader

```
java.lang.Object
  Source.FileReader
```

---

``` public class FileReader extends java.lang.Object ```

This object is designed to simplify the process of reading from a file. It
contains several methods which will allow a developer to easily pull the
desired text from a file.

---

| **Field Summary** | |
| --- | --- |
| `static java.lang.String` | `COMMA_DELIMITED`             Represents the regular expression that will delimit the file by commas |
| `static java.lang.String` | `SPACE_DELIMITED`             Represents the regular expression that will delimit the file by spaces |
| `static java.lang.String` | `TAB_DELIMITED`             Represents the regular expression that will delimit the file by tabs |


| **Constructor Summary** | |
| --- | --- |
| `FileReader(java.io.File file)`             This constructor accepts the path of the file to be opened as a File object, opens it, and prepares the object to be used for reading. |
| `FileReader(java.io.File file, java.lang.String delim)`             This constructor accepts the path of the file to be opened as a File object, opens it, and prepares the object to be used for reading. |
| `FileReader(java.lang.String filename)`             This constructor accepts the path of the file to be opened as a string, opens it, and prepares the object to be used for reading. |
| `FileReader(java.lang.String filename, java.lang.String delim)`             This constructor accepts the path of the file to be opened as a string, opens it, and prepares the object to be used for reading. |


| **Method Summary** | |
| --- | --- |
| `protected  void` | `finalize()`             Closes the file when this object is destroyed. |
| `java.lang.String` | `getCurrentLine()`             This method returns the line that FileReader is currently on. |
| `java.lang.String` | `getNextLine()`              This function returns the next line in the file. |
| `java.lang.String` | `getNextWord()`             This function returns the next word in the line that is currently being read. |
| `int` | `getNumberOfLines()`             Returns the number of lines in the file |
| `int` | `getNumberOfWords()`             Returns the number of words in the current line |
| `int` | `indexOf(java.lang.String needle)`             This function is similar to String's 'indexOf' function, it searches for needle and returns the index of the first character in needle, and -1 if needle is null or the string can't be found in the file. |
| `int` | `indexOf(java.lang.String needle, int startFrom)`             This function is similar to String's 'indexOf' function, it searches for needle and returns the index of the first character in needle, and -1 if needle is null or the string can't be found in the file. |
| `boolean` | `isReady()`             Returns whether or not the file is ready to be read from (usually: if the end of the file has been reached or not) |
| `void` | `reset()`             This function resets the FileReader to the beginning of the file. |
| `void` | `setPosition(int index)`             This method sets the position of this objects BufferedReader to index |
| `java.lang.String` | `substring(int begin, int end)`             This function performs similarly to String's substring function. |
| `java.lang.String` | `toString()`             Returns the entire contents of the file. |

| **Methods inherited from class java.lang.Object** |
| --- |
| `clone, equals, getClass, hashCode, notify, notifyAll, wait, wait, wait` |

| **Field Detail** |
| --- |

### TAB\_DELIMITED

```
public static final java.lang.String TAB_DELIMITED
```

:   Represents the regular expression that will delimit the file by tabs

    **See Also:**: Constant Field Values

---


### COMMA\_DELIMITED

```
public static final java.lang.String COMMA_DELIMITED
```

:   Represents the regular expression that will delimit the file by commas

    **See Also:**: Constant Field Values

---


### SPACE\_DELIMITED

```
public static final java.lang.String SPACE_DELIMITED
```

:   Represents the regular expression that will delimit the file by spaces

    **See Also:**: Constant Field Values


| **Constructor Detail** |
| --- |

### FileReader

```
public FileReader(java.lang.String filename)
           throws java.io.FileNotFoundException,
                  java.io.IOException
```

:   This constructor accepts the path of the file to be opened as a string,
    opens it, and prepares the object to be used for reading.

    **Parameters:**: `filename` - The path of the file to be opened **Throws:**: `java.io.FileNotFoundException` - If the BufferedReader can't find the file: `java.io.IOException` - If an I/O error occurs while reading the first line

---


### FileReader

```
public FileReader(java.lang.String filename,
                  java.lang.String delim)
           throws java.io.FileNotFoundException,
                  java.io.IOException
```

:   This constructor accepts the path of the file to be opened as a string,
    opens it, and prepares the object to be used for reading.

    **Parameters:**: `filename` - The path of the file to be opened: `delim` - The regular expression representing how to delimit the file **Throws:**: `java.io.FileNotFoundException` - If the BufferedReader can't find the file: `java.io.IOException` - If an I/O error occurs while reading the first line

---


### FileReader

```
public FileReader(java.io.File file)
           throws java.io.FileNotFoundException,
                  java.io.IOException
```

:   This constructor accepts the path of the file to be opened as a File
    object, opens it, and prepares the object to be used for reading.

    **Parameters:**: `file` - The File object representing the file to be opened **Throws:**: `java.io.FileNotFoundException` - If the BufferedReader can't find the file: `java.io.IOException` - If an I/O error occurs while reading the first line

---


### FileReader

```
public FileReader(java.io.File file,
                  java.lang.String delim)
           throws java.io.FileNotFoundException,
                  java.io.IOException
```

:   This constructor accepts the path of the file to be opened as a File
    object, opens it, and prepares the object to be used for reading.

    **Parameters:**: `file` - The File object representing the file to be opened: `delim` - The regular expression representing how to delimit the file **Throws:**: `java.io.FileNotFoundException` - If the BufferedReader can't find the file: `java.io.IOException` - If an I/O error occurs while reading the first line


| **Method Detail** |
| --- |

### indexOf

```
public int indexOf(java.lang.String needle)
            throws java.io.FileNotFoundException,
                   java.io.IOException
```

:   This function is similar to String's 'indexOf' function, it searches for
    needle and returns the index of the first character in needle, and -1 if
    needle is null or the string can't be found in the file.

    :   **Parameters:**: `needle` - The string to be found **Returns:**: The index of the first character of needle if it was found, -1 otherwise **Throws:**: `java.io.FileNotFoundException` - If the BufferedReader can't find the file: `java.io.IOException` - If an I/O error occurs while reading from the file

---


### indexOf

```
public int indexOf(java.lang.String needle,
                   int startFrom)
            throws java.io.FileNotFoundException,
                   java.io.IOException
```

:   This function is similar to String's 'indexOf' function, it searches for
    needle and returns the index of the first character in needle, and -1 if
    needle is null or the string can't be found in the file. This method
    starts searching from the index indicated by startFrom

    :   **Parameters:**: `needle` - The string to be found: `startFrom` - The index to start searching from, inclusive **Returns:**: The index of the first character of needle if it was found, -1 otherwise **Throws:**: `java.io.FileNotFoundException` - If the BufferedReader can't find the file: `java.io.IOException` - If an I/O error occurs while reading from the file

---


### getCurrentLine

```
public java.lang.String getCurrentLine()
```

:   This method returns the line that FileReader is currently on.

    :   **Returns:**: The line of the file that is currently being read, null if the end of the file has been reached

---


### getNextLine

```
public java.lang.String getNextLine()
                             throws java.io.IOException
```

:   This function returns the next line in the file. Take note that if
    you have used the getNextWord() function, that the next line ready to be
    read will be the same line that you are getting the words from.  
    Example:  
    Suppose we have a file that has the following as its first line:  
    Hello World!  
    The following snippet of code will result in the following output:  
    FileReader fr = new FileReader(someFile);  
    System.out.println(fr.getNextWord());  
    System.out.println(fr.getNextWord());  
    System.out.println(fr.getNextLine());  
    Output:  
    Hello  
    World!  
    Hello World!  

    :   **Returns:**: The next line in the file, null if the end of the file has been reached **Throws:**: `java.io.IOException` - If an I/O error occurs while reading.

---


### getNextWord

```
public java.lang.String getNextWord()
                             throws java.io.IOException
```

:   This function returns the next word in the line that is currently being
    read. This function uses spaces as tokenizers to separate words.

    :   **Returns:**: The next word in line that is currently being read, null if there are no more words in the line. **Throws:**: `java.io.IOException` - If an I/O error occurs while reading.

---


### getNumberOfWords

```
public int getNumberOfWords()
```

:   Returns the number of words in the current line

    :   **Returns:**: The number of words in the current line

---


### getNumberOfLines

```
public int getNumberOfLines()
                     throws java.io.FileNotFoundException,
                            java.io.IOException
```

:   Returns the number of lines in the file

    :   **Returns:**: The number of lines in the file **Throws:**: `java.io.FileNotFoundException`: `java.io.IOException`

---


### isReady

```
public boolean isReady()
                throws java.io.IOException
```

:   Returns whether or not the file is ready to be read from (usually: if
    the end of the file has been reached or not)

    :   **Returns:**: True if the file is ready, false otherwise **Throws:**: `java.io.IOException` - If an I/O error occurs while checking for readiness

---


### reset

```
public void reset()
           throws java.io.IOException,
                  java.io.FileNotFoundException
```

:   This function resets the FileReader to the beginning of the file.

    :   **Throws:**: `java.io.IOException` - If an I/O error occurs while reading.: `java.io.FileNotFoundException` - If the BufferedReader can't find the file.

---


### setPosition

```
public void setPosition(int index)
                 throws java.io.IOException
```

:   This method sets the position of this objects BufferedReader to index

    :   **Parameters:**: `index` - The position to skip to **Throws:**: `java.io.IOException`

---


### substring

```
public java.lang.String substring(int begin,
                                  int end)
                           throws java.io.FileNotFoundException,
                                  java.io.IOException
```

:   This function performs similarly to String's substring function. It
    returns the characters beginning with begin up to and including the
    character at index end.

    :   **Parameters:**: `begin` - The index of the first character, inclusive.: `end` - The index of the last character, exclusive. **Returns:**: The String representing the characters from begin up to and including end. **Throws:**: `java.io.FileNotFoundException` - If the BufferedReader can't find the file.: `java.io.IOException` - If an I/O error occurs while reading.: `java.lang.IndexOutOfBoundsException` - If begin is greater than end, or if the specified indices do not exist.

---


### toString

```
public java.lang.String toString()
```

:   Returns the entire contents of the file.

    :   **Overrides:**: `toString` in class `java.lang.Object`
    :   **Returns:**: The entire contents of the file as a String.

---


### finalize

```
protected void finalize()
                 throws java.lang.Throwable
```

:   Closes the file when this object is destroyed.

    :   **Overrides:**: `finalize` in class `java.lang.Object`
    :   **Throws:**: `java.lang.Throwable`


---


|  |  |  |  |  |  |  |  |  |  |
| --- | --- | --- | --- | --- | --- | --- | --- | --- | --- |
| |  |  |  |  |  |  |  | | --- | --- | --- | --- | --- | --- | --- | | **Package** | **Class** | **Use** | **Tree** | **Deprecated** | **Index** | **Help** | | |  |
| **PREV CLASS**   NEXT CLASS | **FRAMES**    **NO FRAMES**     **All Classes** |
| SUMMARY: NESTED | FIELD | CONSTR | METHOD | DETAIL: FIELD | CONSTR | METHOD |


---
