# Supplementary material for: Predicted protein-protein interactions in the moss Physcomitrella patens: a new bioinformatic resource
Source: BMC Bioinformatics. 2015 Mar 16;16(1):89. doi: 10.1186/s12859-015-0524-1 (PMC4384322; doi:10.1186/s12859-015-0524-1)
Supplement: Additional file 1: — Software package used in generating the interactome from databases. [file 12859_2015_524_MOESM1_ESM.zip › MySQL_Importer_v1/javadoc/Source/DataImportGUI.html]

DataImportGUI


---


|  |  |  |  |  |  |  |  |  |  |
| --- | --- | --- | --- | --- | --- | --- | --- | --- | --- |
| |  |  |  |  |  |  |  | | --- | --- | --- | --- | --- | --- | --- | | **Package** | **Class** | **Use** | **Tree** | **Deprecated** | **Index** | **Help** | | |  |
| **PREV CLASS**   **NEXT CLASS** | **FRAMES**    **NO FRAMES**     **All Classes** |
| SUMMARY: NESTED | FIELD | CONSTR | METHOD | DETAIL: FIELD | CONSTR | METHOD |


---


## Source Class DataImportGUI

```
java.lang.Object
  java.awt.Component
      java.awt.Container
          java.awt.Window
              java.awt.Frame
                  javax.swing.JFrame
                      Source.DataImportGUI
```

**All Implemented Interfaces:**: java.awt.image.ImageObserver, java.awt.MenuContainer, java.io.Serializable, javax.accessibility.Accessible, javax.swing.RootPaneContainer, javax.swing.WindowConstants

---

``` public class DataImportGUI extends javax.swing.JFrame ```

This is the driver class for the MySQL DataImport program. It generates a
GUI that allows a user to specify an output and input file as well as
specify whether the file is comma-separated or tab-separated. The program
will then create a SQL script file consisting of a series of INSERT
statements which will place the data in the input file into a database which
is named the same as the input file.

**See Also:**: Serialized Form

---

| **Nested Class Summary** | |
| --- | --- |

| **Nested classes/interfaces inherited from class javax.swing.JFrame** |
| --- |
| `javax.swing.JFrame.AccessibleJFrame` |

| **Nested classes/interfaces inherited from class java.awt.Frame** |
| --- |
| `java.awt.Frame.AccessibleAWTFrame` |

| **Nested classes/interfaces inherited from class java.awt.Window** |
| --- |
| `java.awt.Window.AccessibleAWTWindow` |

| **Nested classes/interfaces inherited from class java.awt.Container** |
| --- |
| `java.awt.Container.AccessibleAWTContainer` |

| **Nested classes/interfaces inherited from class java.awt.Component** |
| --- |
| `java.awt.Component.AccessibleAWTComponent, java.awt.Component.BaselineResizeBehavior, java.awt.Component.BltBufferStrategy, java.awt.Component.FlipBufferStrategy` |


| **Field Summary** | |
| --- | --- |

| **Fields inherited from class javax.swing.JFrame** |
| --- |
| `accessibleContext, EXIT_ON_CLOSE, rootPane, rootPaneCheckingEnabled` |

| **Fields inherited from class java.awt.Frame** |
| --- |
| `CROSSHAIR_CURSOR, DEFAULT_CURSOR, E_RESIZE_CURSOR, HAND_CURSOR, ICONIFIED, MAXIMIZED_BOTH, MAXIMIZED_HORIZ, MAXIMIZED_VERT, MOVE_CURSOR, N_RESIZE_CURSOR, NE_RESIZE_CURSOR, NORMAL, NW_RESIZE_CURSOR, S_RESIZE_CURSOR, SE_RESIZE_CURSOR, SW_RESIZE_CURSOR, TEXT_CURSOR, W_RESIZE_CURSOR, WAIT_CURSOR` |

| **Fields inherited from class java.awt.Component** |
| --- |
| `BOTTOM_ALIGNMENT, CENTER_ALIGNMENT, LEFT_ALIGNMENT, RIGHT_ALIGNMENT, TOP_ALIGNMENT` |

| **Fields inherited from interface javax.swing.WindowConstants** |
| --- |
| `DISPOSE_ON_CLOSE, DO_NOTHING_ON_CLOSE, HIDE_ON_CLOSE` |

| **Fields inherited from interface java.awt.image.ImageObserver** |
| --- |
| `ABORT, ALLBITS, ERROR, FRAMEBITS, HEIGHT, PROPERTIES, SOMEBITS, WIDTH` |


| **Constructor Summary** | |
| --- | --- |
| `DataImportGUI()`             Creates new form DataImportGUI |


| **Method Summary** | |
| --- | --- |
| `static void` | `main(java.lang.String[] args)` |
| `void` | `updateProgress(double p)` |

| **Methods inherited from class javax.swing.JFrame** |
| --- |
| `addImpl, createRootPane, frameInit, getAccessibleContext, getContentPane, getDefaultCloseOperation, getGlassPane, getGraphics, getJMenuBar, getLayeredPane, getRootPane, getTransferHandler, isDefaultLookAndFeelDecorated, isRootPaneCheckingEnabled, paramString, processWindowEvent, remove, repaint, setContentPane, setDefaultCloseOperation, setDefaultLookAndFeelDecorated, setGlassPane, setIconImage, setJMenuBar, setLayeredPane, setLayout, setRootPane, setRootPaneCheckingEnabled, setTransferHandler, update` |

| **Methods inherited from class java.awt.Frame** |
| --- |
| `addNotify, getCursorType, getExtendedState, getFrames, getIconImage, getMaximizedBounds, getMenuBar, getState, getTitle, isResizable, isUndecorated, remove, removeNotify, setCursor, setExtendedState, setMaximizedBounds, setMenuBar, setResizable, setState, setTitle, setUndecorated` |

| **Methods inherited from class java.awt.Window** |
| --- |
| `addPropertyChangeListener, addPropertyChangeListener, addWindowFocusListener, addWindowListener, addWindowStateListener, applyResourceBundle, applyResourceBundle, createBufferStrategy, createBufferStrategy, dispose, getBufferStrategy, getFocusableWindowState, getFocusCycleRootAncestor, getFocusOwner, getFocusTraversalKeys, getGraphicsConfiguration, getIconImages, getInputContext, getListeners, getLocale, getModalExclusionType, getMostRecentFocusOwner, getOwnedWindows, getOwner, getOwnerlessWindows, getToolkit, getWarningString, getWindowFocusListeners, getWindowListeners, getWindows, getWindowStateListeners, hide, isActive, isAlwaysOnTop, isAlwaysOnTopSupported, isFocusableWindow, isFocusCycleRoot, isFocused, isLocationByPlatform, isShowing, pack, postEvent, processEvent, processWindowFocusEvent, processWindowStateEvent, removeWindowFocusListener, removeWindowListener, removeWindowStateListener, reshape, setAlwaysOnTop, setBounds, setBounds, setCursor, setFocusableWindowState, setFocusCycleRoot, setIconImages, setLocationByPlatform, setLocationRelativeTo, setMinimumSize, setModalExclusionType, setSize, setSize, setVisible, show, toBack, toFront` |

| **Methods inherited from class java.awt.Container** |
| --- |
| `add, add, add, add, add, addContainerListener, applyComponentOrientation, areFocusTraversalKeysSet, countComponents, deliverEvent, doLayout, findComponentAt, findComponentAt, getAlignmentX, getAlignmentY, getComponent, getComponentAt, getComponentAt, getComponentCount, getComponents, getComponentZOrder, getContainerListeners, getFocusTraversalPolicy, getInsets, getLayout, getMaximumSize, getMinimumSize, getMousePosition, getPreferredSize, insets, invalidate, isAncestorOf, isFocusCycleRoot, isFocusTraversalPolicyProvider, isFocusTraversalPolicySet, layout, list, list, locate, minimumSize, paint, paintComponents, preferredSize, print, printComponents, processContainerEvent, remove, removeAll, removeContainerListener, setComponentZOrder, setFocusTraversalKeys, setFocusTraversalPolicy, setFocusTraversalPolicyProvider, setFont, transferFocusBackward, transferFocusDownCycle, validate, validateTree` |

| **Methods inherited from class java.awt.Component** |
| --- |
| `action, add, addComponentListener, addFocusListener, addHierarchyBoundsListener, addHierarchyListener, addInputMethodListener, addKeyListener, addMouseListener, addMouseMotionListener, addMouseWheelListener, bounds, checkImage, checkImage, coalesceEvents, contains, contains, createImage, createImage, createVolatileImage, createVolatileImage, disable, disableEvents, dispatchEvent, enable, enable, enableEvents, enableInputMethods, firePropertyChange, firePropertyChange, firePropertyChange, firePropertyChange, firePropertyChange, firePropertyChange, firePropertyChange, firePropertyChange, firePropertyChange, getBackground, getBaseline, getBaselineResizeBehavior, getBounds, getBounds, getColorModel, getComponentListeners, getComponentOrientation, getCursor, getDropTarget, getFocusListeners, getFocusTraversalKeysEnabled, getFont, getFontMetrics, getForeground, getHeight, getHierarchyBoundsListeners, getHierarchyListeners, getIgnoreRepaint, getInputMethodListeners, getInputMethodRequests, getKeyListeners, getLocation, getLocation, getLocationOnScreen, getMouseListeners, getMouseMotionListeners, getMousePosition, getMouseWheelListeners, getName, getParent, getPeer, getPropertyChangeListeners, getPropertyChangeListeners, getSize, getSize, getTreeLock, getWidth, getX, getY, gotFocus, handleEvent, hasFocus, imageUpdate, inside, isBackgroundSet, isCursorSet, isDisplayable, isDoubleBuffered, isEnabled, isFocusable, isFocusOwner, isFocusTraversable, isFontSet, isForegroundSet, isLightweight, isMaximumSizeSet, isMinimumSizeSet, isOpaque, isPreferredSizeSet, isValid, isVisible, keyDown, keyUp, list, list, list, location, lostFocus, mouseDown, mouseDrag, mouseEnter, mouseExit, mouseMove, mouseUp, move, nextFocus, paintAll, prepareImage, prepareImage, printAll, processComponentEvent, processFocusEvent, processHierarchyBoundsEvent, processHierarchyEvent, processInputMethodEvent, processKeyEvent, processMouseEvent, processMouseMotionEvent, processMouseWheelEvent, removeComponentListener, removeFocusListener, removeHierarchyBoundsListener, removeHierarchyListener, removeInputMethodListener, removeKeyListener, removeMouseListener, removeMouseMotionListener, removeMouseWheelListener, removePropertyChangeListener, removePropertyChangeListener, repaint, repaint, repaint, requestFocus, requestFocus, requestFocusInWindow, requestFocusInWindow, resize, resize, setBackground, setComponentOrientation, setDropTarget, setEnabled, setFocusable, setFocusTraversalKeysEnabled, setForeground, setIgnoreRepaint, setLocale, setLocation, setLocation, setMaximumSize, setName, setPreferredSize, show, size, toString, transferFocus, transferFocusUpCycle` |

| **Methods inherited from class java.lang.Object** |
| --- |
| `clone, equals, finalize, getClass, hashCode, notify, notifyAll, wait, wait, wait` |

| **Methods inherited from interface java.awt.MenuContainer** |
| --- |
| `getFont, postEvent` |

| **Constructor Detail** |
| --- |

### DataImportGUI

```
public DataImportGUI()
```

:   Creates new form DataImportGUI


| **Method Detail** |
| --- |

### updateProgress

```
public void updateProgress(double p)
```

---


### main

```
public static void main(java.lang.String[] args)
```

:   **Parameters:**: `args` - the command line arguments


---


|  |  |  |  |  |  |  |  |  |  |
| --- | --- | --- | --- | --- | --- | --- | --- | --- | --- |
| |  |  |  |  |  |  |  | | --- | --- | --- | --- | --- | --- | --- | | **Package** | **Class** | **Use** | **Tree** | **Deprecated** | **Index** | **Help** | | |  |
| **PREV CLASS**   **NEXT CLASS** | **FRAMES**    **NO FRAMES**     **All Classes** |
| SUMMARY: NESTED | FIELD | CONSTR | METHOD | DETAIL: FIELD | CONSTR | METHOD |


---
