# Supplementary material for: Predicted protein-protein interactions in the moss Physcomitrella patens: a new bioinformatic resource
Source: BMC Bioinformatics. 2015 Mar 16;16(1):89. doi: 10.1186/s12859-015-0524-1 (PMC4384322; doi:10.1186/s12859-015-0524-1)
Supplement: Additional file 1: — Software package used in generating the interactome from databases. [file 12859_2015_524_MOESM1_ESM.zip › MySQL_Importer_v1/javadoc/Source/DataImport.html]

DataImport


---


|  |  |  |  |  |  |  |  |  |  |
| --- | --- | --- | --- | --- | --- | --- | --- | --- | --- |
| |  |  |  |  |  |  |  | | --- | --- | --- | --- | --- | --- | --- | | **Package** | **Class** | **Use** | **Tree** | **Deprecated** | **Index** | **Help** | | |  |
| PREV CLASS   **NEXT CLASS** | **FRAMES**    **NO FRAMES**     **All Classes** |
| SUMMARY: NESTED | FIELD | CONSTR | METHOD | DETAIL: FIELD | CONSTR | METHOD |


---


## Source Class DataImport

```
java.lang.Object
  java.lang.Thread
      Source.DataImport
```

**All Implemented Interfaces:**: java.lang.Runnable

---

``` public class DataImport extends java.lang.Thread ```

This object will take data from a file (CSV or TSV), and create a MySQL
script that will be able to import the data into a database. The first line
of the file must be the names of the fields (columns) seperated by tabs (if
the file is TSV) or commas (if the file is CSV).

---

| **Nested Class Summary** | |
| --- | --- |

| **Nested classes/interfaces inherited from class java.lang.Thread** |
| --- |
| `java.lang.Thread.State, java.lang.Thread.UncaughtExceptionHandler` |


| **Field Summary** | |
| --- | --- |
| `static int` | `CSV`             Represents the comma-seperated delimited type |
| `static int` | `TSV`             Represents the tab-seperated delimited type |

| **Fields inherited from class java.lang.Thread** |
| --- |
| `MAX_PRIORITY, MIN_PRIORITY, NORM_PRIORITY` |


| **Constructor Summary** | |
| --- | --- |
| `DataImport(java.io.File inFile, java.io.File outFile, int delim, DataImportGUI gui)`             Creates a new DataImport thread |


| **Method Summary** | |
| --- | --- |
| `void` | `close()`             Properly close the object |
| `void` | `run()` |

| **Methods inherited from class java.lang.Thread** |
| --- |
| `activeCount, checkAccess, countStackFrames, currentThread, destroy, dumpStack, enumerate, getAllStackTraces, getContextClassLoader, getDefaultUncaughtExceptionHandler, getId, getName, getPriority, getStackTrace, getState, getThreadGroup, getUncaughtExceptionHandler, holdsLock, interrupt, interrupted, isAlive, isDaemon, isInterrupted, join, join, join, resume, setContextClassLoader, setDaemon, setDefaultUncaughtExceptionHandler, setName, setPriority, setUncaughtExceptionHandler, sleep, sleep, start, stop, stop, suspend, toString, yield` |

| **Methods inherited from class java.lang.Object** |
| --- |
| `clone, equals, finalize, getClass, hashCode, notify, notifyAll, wait, wait, wait` |

| **Field Detail** |
| --- |

### CSV

```
public static final int CSV
```

:   Represents the comma-seperated delimited type

    **See Also:**: Constant Field Values

---


### TSV

```
public static final int TSV
```

:   Represents the tab-seperated delimited type

    **See Also:**: Constant Field Values


| **Constructor Detail** |
| --- |

### DataImport

```
public DataImport(java.io.File inFile,
                  java.io.File outFile,
                  int delim,
                  DataImportGUI gui)
           throws java.io.IOException,
                  java.io.FileNotFoundException
```

:   Creates a new DataImport thread

    **Parameters:**: `inFile` - The file containing the data to be imported: `outFile` - The file to save the output to: `delim` - The delimited type of the file, use either DataImport.CSV or DataImport.TSV: `gui` - The GUI that created this object **Throws:**: `java.io.IOException` - If anI/O error occurs while reading to/from the file: `java.io.FileNotFoundException` - If either the input or output file can't be found


| **Method Detail** |
| --- |

### run

```
public void run()
```

:   **Specified by:**: `run` in interface `java.lang.Runnable` **Overrides:**: `run` in class `java.lang.Thread`

---


### close

```
public void close()
```

:   Properly close the object


---


|  |  |  |  |  |  |  |  |  |  |
| --- | --- | --- | --- | --- | --- | --- | --- | --- | --- |
| |  |  |  |  |  |  |  | | --- | --- | --- | --- | --- | --- | --- | | **Package** | **Class** | **Use** | **Tree** | **Deprecated** | **Index** | **Help** | | |  |
| PREV CLASS   **NEXT CLASS** | **FRAMES**    **NO FRAMES**     **All Classes** |
| SUMMARY: NESTED | FIELD | CONSTR | METHOD | DETAIL: FIELD | CONSTR | METHOD |


---
