# Supplementary material for: Predicted protein-protein interactions in the moss Physcomitrella patens: a new bioinformatic resource
Source: BMC Bioinformatics. 2015 Mar 16;16(1):89. doi: 10.1186/s12859-015-0524-1 (PMC4384322; doi:10.1186/s12859-015-0524-1)
Supplement: Additional file 1: — Software package used in generating the interactome from databases. [file 12859_2015_524_MOESM1_ESM.zip › MySQL_Importer_v1/javadoc/Source/class-use/DataImport.html]

Uses of Class Source.DataImport


---


|  |  |  |  |  |  |  |  |  |  |
| --- | --- | --- | --- | --- | --- | --- | --- | --- | --- |
| |  |  |  |  |  |  |  | | --- | --- | --- | --- | --- | --- | --- | | **Package** | **Class** | **Use** | **Tree** | **Deprecated** | **Index** | **Help** | | |  |
| PREV   NEXT | **FRAMES**    **NO FRAMES**     **All Classes** |


---


## **Uses of Class Source.DataImport**

No usage of Source.DataImport

---


|  |  |  |  |  |  |  |  |  |  |
| --- | --- | --- | --- | --- | --- | --- | --- | --- | --- |
| |  |  |  |  |  |  |  | | --- | --- | --- | --- | --- | --- | --- | | **Package** | **Class** | **Use** | **Tree** | **Deprecated** | **Index** | **Help** | | |  |
| PREV   NEXT | **FRAMES**    **NO FRAMES**     **All Classes** |


---
